# KK-LC-1, a biomarker for prognosis of immunotherapy for primary liver cancer

**DOI:** 10.1186/s12885-024-12586-y

**Published:** 2024-07-07

**Authors:** Sihui Zhu, Yuncheng Jin, Mingzhen Zhou, Lin Li, Xueru Song, Xinyu Su, Baorui Liu, Jie Shen

**Affiliations:** 1 Comprehensive Cancer Centre, Department of Oncology, Nanjing Drum Tower Hospital, The Affiliated Hospital of Nanjing University Medical School, Nanjing, China; 2 Comprehensive Cancer Centre of Nanjing international Hospital, Medical School of Nanjing University, Nanjing, China; 3 Clinical Cancer Institute of Nanjing University, Nanjing, China; 4 Department of Pathologyof Nanjing Drum Tower Hospital, The Affiliated Hospital of Nanjing University Medical School, Nanjing, China

**Keywords:** Immunotherapy, Hepatocellular carcinoma, Biomarkers

## Abstract

**Purpose:**

There is mounting evidence that patients with liver cancer can benefit from Immune checkpoint inhibitors. However, due to the high cost and low efficacy, we aimed to explore new biomarkers for predicting the efficacy of immunotherapy.

**Methods:**

Specimens and medical records of liver cancer patients treated at Drum Tower Hospital of Nanjing University were collected, and the expression of Kita-Kyushu lung cancer antigen-1 (KK-LC-1) in tissues as well as the corresponding antibodies in serum were examined to find biomarkers related to the prognosis of immunotherapy and to explore its mechanism in the development of liver cancer.

**Results:**

KK-LC-1 expression was found to be 34.4% in histopathological specimens from 131 patients and was significantly correlated with Foxp3 expression (*P* = 0.0356). The expression of Foxp3 in the tissues of 24 patients who received immunotherapy was significantly correlated with overall survival (OS) (*P* = 0.0247), and there was also a tendency for prolonged OS in patients with high expression of KK-LC-1. In addition, the expression of KK-LC-1 antibody in the serum of patients who received immunotherapy with a first efficacy evaluation of stable disease (SD) was significantly higher than those with partial response (PR) (*P* = 0.0413).

**Conclusions:**

Expression of KK-LC-1 in both tissues and serum has been shown to correlate with the prognosis of patients treated with immunotherapy, and KK-LC-1 is a potential therapeutic target for oncological immunotherapy.

**Supplementary Information:**

The online version contains supplementary material available at 10.1186/s12885-024-12586-y.

## Introduction

The incidence of liver cancer has been rising all over the world [1]. At present, it has been the second leading cause of cancer-related death after lung cancer [2]. In recent years, immunotherapy has become a hot spot in tumor treatment. Immune checkpoint inhibitors (ICIs) such as programmed cell death receptor 1 (PD-1), programmed cell death-ligand 1 (PD-L1), and Cytotoxic T lymphocyte associate protein-4 (CTLA-4) monoclonal antibodies have been shown an effective way to reduce tumor size, slow tumor progression, and prolonging overall survival of patients [3, 4]. However, the efficiency of immunotherapy is only 20% [5], and the incidence of immune-related adverse events (irAEs) ranges from 54–76% [6]. Some patients may also experience hyper-progressive disease during the process [7, 8]. Therefore, exploring biomarkers for accurately predicting the efficacy of immunotherapy is of great significance in individualized treatment of liver cancer.

PD-L1 [9], microsatellite instability-high (MSI-H) [10] and tumor mutation burden (TMB) [11] are commonly used biomarkers in clinical practice. In liver cancer, the expression of PD-L1 is low, less than 20% [12], and it has been verified that the baseline expression of PD-L1 is independent of prognosis [13]. The expression of patients with MSI-H is ranged from 0.2 to 3%, and the proportion of patients with TMB higher than 10 mutation/Mb is ranged from 0.8–5% [14]. To date, there is still no predictive biomarker for the immunotherapy of liver cancer.

Cancer-testis Antigen (CTA) is a class of tumor-associated antigen (TAA), which is expressed in normal testis tissue and various tumor tissues [15]. Previous study reported that CTA is a potential target of tumor immunotherapy and is related to high TMB [5]. Some CTAs have oncogenic functions, including promoting tumor growth, evading apoptosis, and inducing invasion and metastasis [16], and thus is considered to be a specific marker of prognosis [17]. Kita-Kyushu lung cancer antigen-1 (KK-LC-1) is a kind of CTA, which was firstly identified in lung cancer tissue by the team of Takashi Fukuyama [18]. It is also abnormally expressed in gastric cancer [19], breast cancer [20], and liver cancer [21]. In animal models, it has been verified that KK-LC-1 can promote tumor development in vivo [21].

In this study, we analyzed the relationship between the expression of KK-LC-1 and the prognosis of liver cancer patients by detecting the expression of KK-LC-1 and its corresponding antibodies in serum, aiming to find biomarkers that can predict the prognosis of immunotherapy in liver cancer patients, and further explore its mechanism.

## Materials and methods

### Patients

A total of 131 paraffin-embedded tissue specimens that underwent surgical resection or pathological biopsy for liver cancer in Nanjing Drum Tower Hospital from April 2018 to December 2020, were archived in the pathology department and clearly diagnosed as primary hepatocellular carcinoma or intrahepatic cholangiocarcinoma were collected retrospectively. Among them, 23 patients had received single-agent immunotherapy, while 1 patient with ICC had undergone targeted combination immunotherapy. All patients provided full consent for this study. The Ethics Committee of Nanjing Drum Tower Hospital approved the collection of samples and medical records.

### Immunohistochemistry

Paraffin-embedded tissues were cut into 3 microns, attached to slides, and then dewaxed and hydrated. Antigen repair was performed by boiling the slides with antigen repair solution. Mouse clonal antibody KK-LC-1 (Thermo Fisher, USA, 1:400) or rabbit-derived monoclonal antibody Foxp3 (Jinqiao Biotechnology Co., Ltd., China, 1:100) were added as the primary antibody, respectively, and the coloration was developed with DAB solution.The staining site of KK-LC-1 was located in the cell membrane of the tumor cells, and the positive staining of Foxp3 molecules was localized in the nesting region TILs.

The immunohistochemical results were observed by two experienced pathologists by double-blind method. Four high magnification fields were randomly selected, and KK-LC-1 immunohistochemical staining scores were calculated based on the percentage of positive cells and the intensity of positive cell staining: (1) 0 points for positive cells < 5%, 1 point for 5-24%, 2 points for 25-49%, 3 points for 50-75%, and 4 points for > 75%; (2) 0 points for no staining, 1 point for light yellow, 2 point for brownish yellow, and 3 point for brownish brown. Then the value corresponding to the intensity of staining × percentage of positive cells was used as the score. We define 0–4 point as low expression of KK-LC-1 and 5–12 point as high expression. Foxp3 expression was scored as the number of positive cells found in the visual field after high magnification (HPF400).

### Cell lines and cell culture

The hepatocellular carcinoma cell lines sk-hep-1 were provided by the Laboratory of Cancer Center, Drum Tower Hospital, School of Medicine, Nanjing University. Cells were cultured in 1640 medium containing 10% fetal bovine serum (FBS), and incubated in a cell incubator at 37 °C with 5% CO_2_ and saturated humidity, passaged every 2–3 days.

### Construction of cell lines with different expression of KK-LC-1

The sk-hep-1-KK-LC-1 and sk-hep-1-con cell lines were constructed by co-incubating sk-hep-1 with lentivirus expressing and not expressing KK-LC-1. The infection efficiency was verified by cellular immunochemical techniques and flow analysis (Supplementary Fig. 1).

### T-cell-mediated tumor cell killing assay

PBMCs (Peripheral blood mononuclear cells) from volunteers were extracted. After pulsing with the predicted and synthesized KK-LC-1 antigenic peptide, the expression of γ-IFN in the supernatant was detected by cytometric bead array (CBA). Followed by grouping the stimulated reactive T cells with CFSE-labeled sk-hep-1-KK-LC-1 and sk-hep-1-con tumor cells according to the predetermined efficacy target ratio. After 16 h, the tumor cells were collected and analyzed by flow cytometry after staining with PI.

### Differentially expressed genes and enrichment analysis

RNAs in the samples was extracted. RNAs differential expression analysis was performed by DESeq2 software between two different groups. The genes with the parameter of false discovery rate (FDR) below 0.05 and absolute fold change ≥ 2 were considered differentially expressed genes. Gene ontology (GO) functional enrichment and Kyoto Encyclopedia of Genes and Genomes (KEGG) pathway were performed using the “clusterProfiler” R package. *P* value < 0.05 was considered the criterion for statistical significance.

### Statistical methods

Analysis was performed using Prism 7 software and the results were plotted. The relationship between KK-LC-1 expression and Foxp3 expression was analyzed by the χ2 test, and antibody expression in serum was logarithmically analyzed by t-test. Kaplan-Meier survival curves were plotted according to the expression of KK-LC-1, and log-rank test was performed between groups, *P* < 0. 05 was considered a statistical difference.

## Result

### Expression of KK-LC-1 and its potential prognostic role in liver cancer

**Fig. 1:**
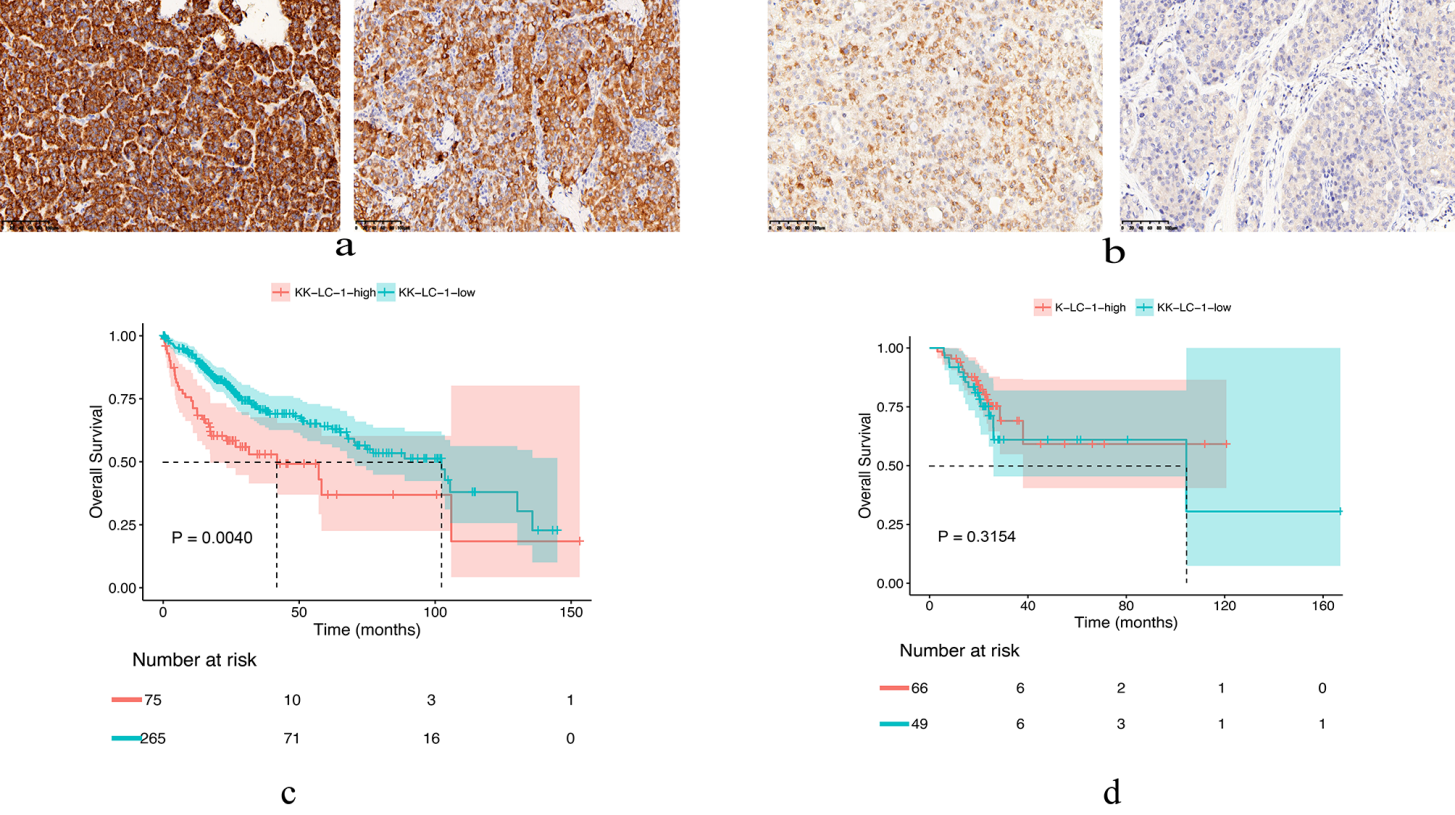
**a**: High expression of KK-LC-1. **b**: Low expression of KK-LC-1. **c**: Searching the TCGA database and plotting survival curves based on KK-LC-1 expression, patients with high KK-LC-1 expression had lower OS than those with low KK-LC-1 expression (*P* = 0.0040). **d**: Relationship between KK-LC-1 expression level and OS in patients

We detected the expression of KK-LC-1 in the tumor tissues of liver cancer patients by immunohistochemistry (IHC) and found that 34.4% (45/131) of them showed a high expression of KK-LC-1 (Fig. 1a, b). This showed great research value for predicting clinical efficacy in liver cancer.

Then, we collected 340 hepatocellular carcinoma (HCC) patients from the TCGA database and divided them into high expression group and low expression group according to the median expression of KK-LC-1. Statistical analysis showed that patients with high KK-LC-1 expression had a shorter overall survival (OS) (*P* = 0.0040) than those with low KK-LC-1 expression (Fig. 1c).

In our cohort, we collected the information of OS and progression free survival (PFS) from 115 patients, and also divided them into high expression group and low expression group according to the expression of KK-LC-1. Statistical analysis showed that the expression of KK-LC-1 in tissues was not associated with OS and PFS of patients (*P* = 0.3145 and *P* = 0.9628, respectively) (Fig. 1d and Supplementary Fig. 2).

### Expression of KK-LC-1 is associated with OS in patients received immunotherapy

**Fig. 2:**
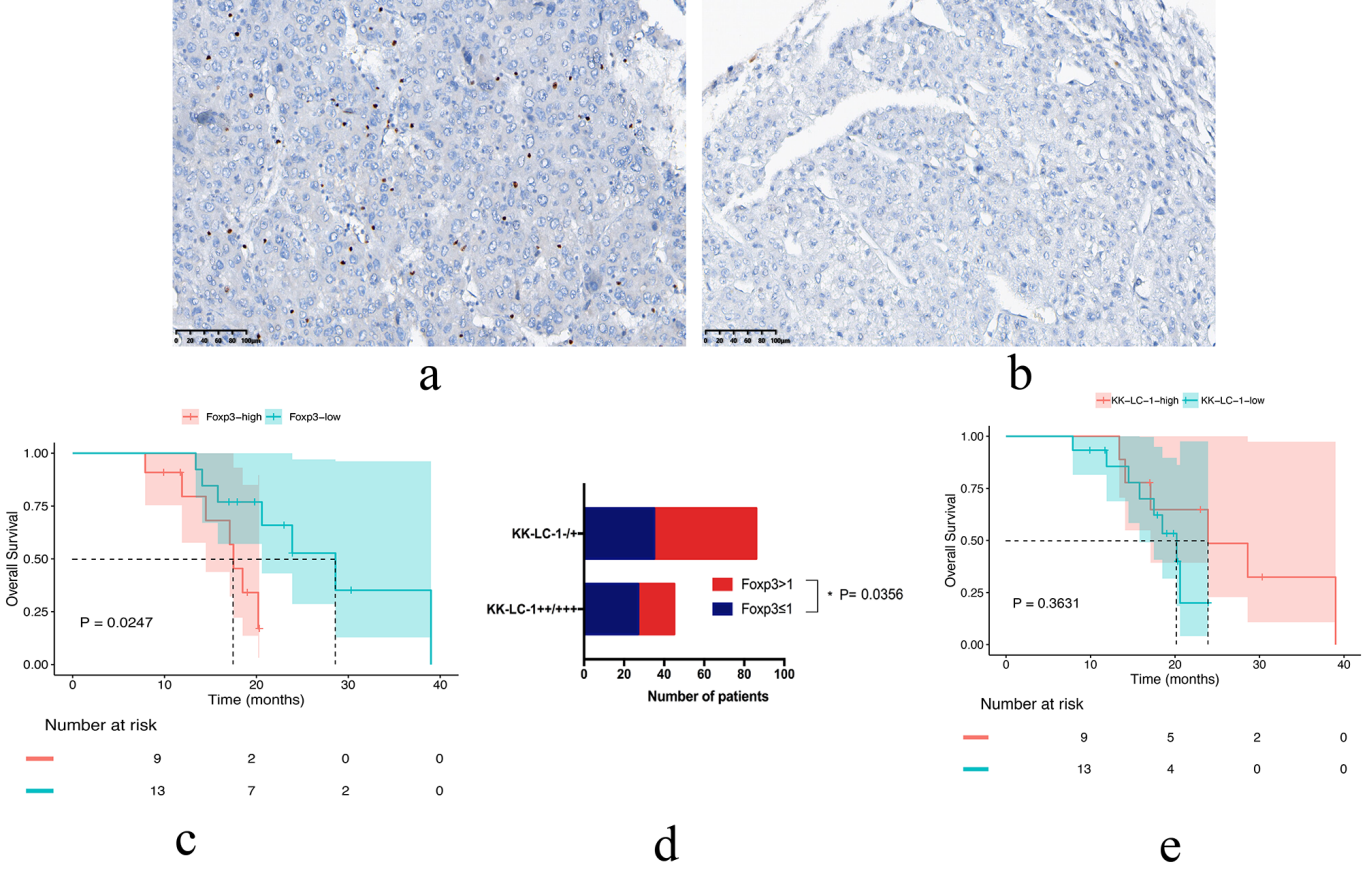
**a**: High expression of Foxp3. **b**: Low expression of Foxp3. **c**: Relationship between Foxp3 expression levels and the OS in patients. **d**: KK-LC-1 showed a significant negative correlation with Foxp3 expression. e: Relationship between KK-LC-1 expression levels and the OS in patients

We also examined Foxp3 expression in tissues (Fig. 2a, b). When the expression of Foxp3 is less than 1/400 HPF, it is considered as high expression. Our results showed that there were 62 patients (47.3%) with low Foxp3 expression and 69 (52.7%) patients with high Foxp3 expression. There were 24 patients who both had complete case information and received immunotherapy. Our results showed that the expression of Foxp3 in their tissues was significantly associated with OS (*P* = 0.0247) (Fig. 2c). Although patients with low Foxp3 expression had a tendncy for longer PFS, there was no statistical difference (*P* = 0.1570) (Supplementary Fig. 3a). Further analysis revealed that the percentage of Foxp3 high expression was significantly lower in tissue specimens with high KK-LC-1 expression than that in tissue specimens with low KK-LC-1 expression (*P* = 0.0356) (Fig. 2d). We observed a tendency for prolonged OS in patients with high KK-LC-1 expression (*P* = 0.3631) (Fig. 2e), and there was no correlation between PFS and KK-LC-1 expression (Supplementary Fig. 3b).

Seventeen of all 24 patients showed low expression of Foxp3 and high expression of KK-LC-1, and their OS tend to be prolonged compared to others (Supplementary Fig. 3c-f).

### The expression of KK-LC-1 antibody changed after patients received immunotherapy

**Fig. 3:**
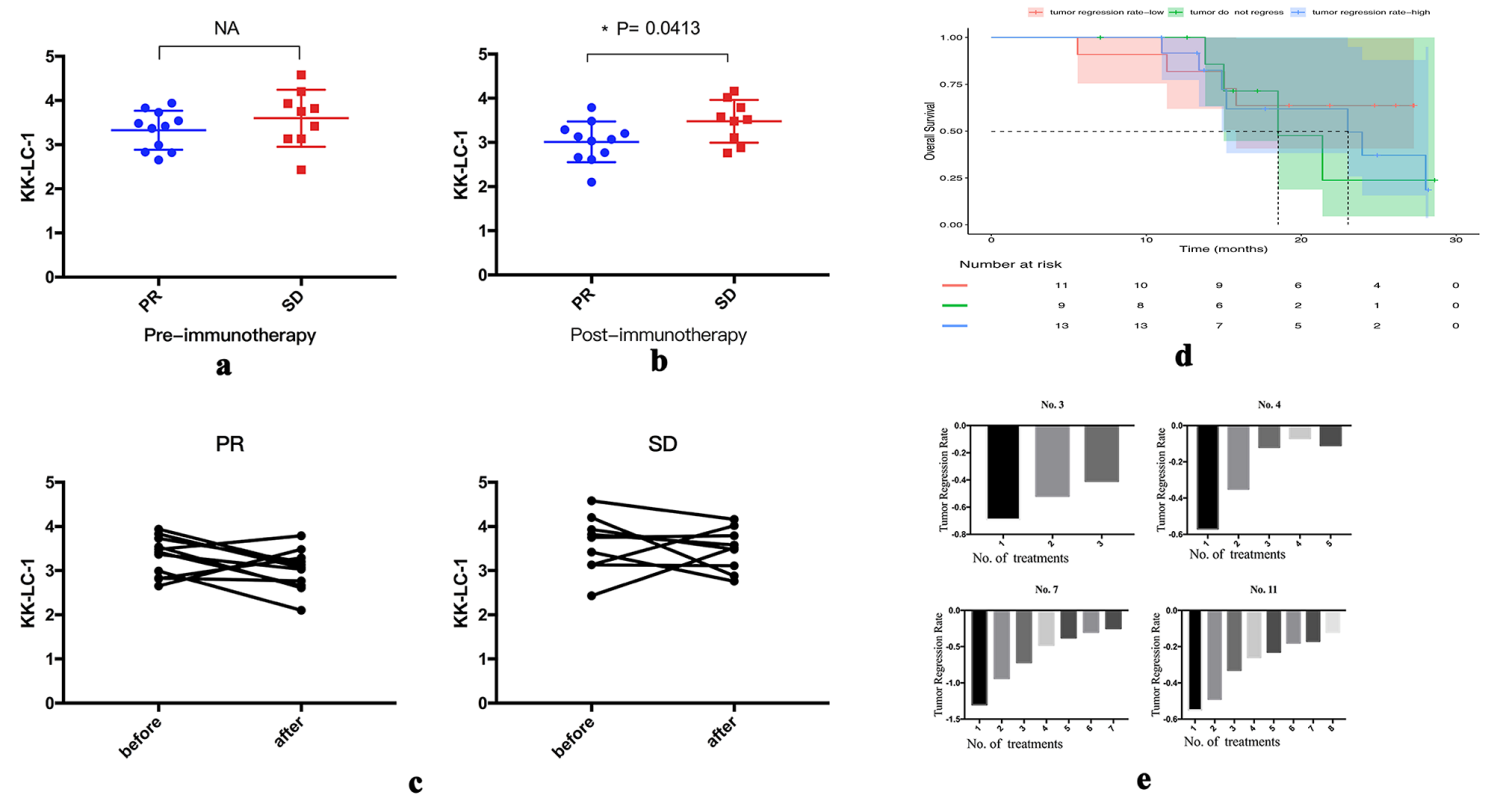
**a**: The relationship between the expression of KK-LC-1 antibody with the efficacy of immunotherapy before this treatment. **b**: The relationship between the expression of KK-LC-1 antibody with the efficacy of immunotherapy after this treatment. **c**: (1) Changes in KK-LC-1 antibody expression before and after immunotherapy in patients with PR. (2) Changes in KK-LC-1 antibody expression before and after immunotherapy in patients with SD. **d**: Relationship between tumor regression rate and patient survival. **e**. The rate of tumor regression in patients tends to decrease in absolute value over time

The expression of serum KK-LC-1 was tested in 20 patients receiving immunotherapy. Of the 20 patients, 11 were evaluated as PR (partial response) and 9 were evaluated as SD (stable disease). Statistical analysis showed that there was no significant association between immunotherapy efficacy and the expression of KK-LC-1 (Fig. 3a). However, at the first efficacy evaluation, with patients in the SD group showing significantly higher expression than those in the PR group (*P* = 0.0413) (Fig. 3b). In the PR group, the expression of KK-LC-1 tended to decrease after receiving immunotherapy. Based on the imaging data of HCC patients, we found that the tumor regression rate at the first review did not correlate with patient survival (Fig. 3d). However, we plotted the tumor regression rate of each patient at each review as a waterfall plot and found that the absolute value of the tumor regression rate gradually decreased as the treatment continued (Fig. 3e).

### KK-LC-1 antigenic peptide is immunogenic and reactive T cells can specifically kill tumor cells with high KK-LC-1 expression

**Fig. 4:**
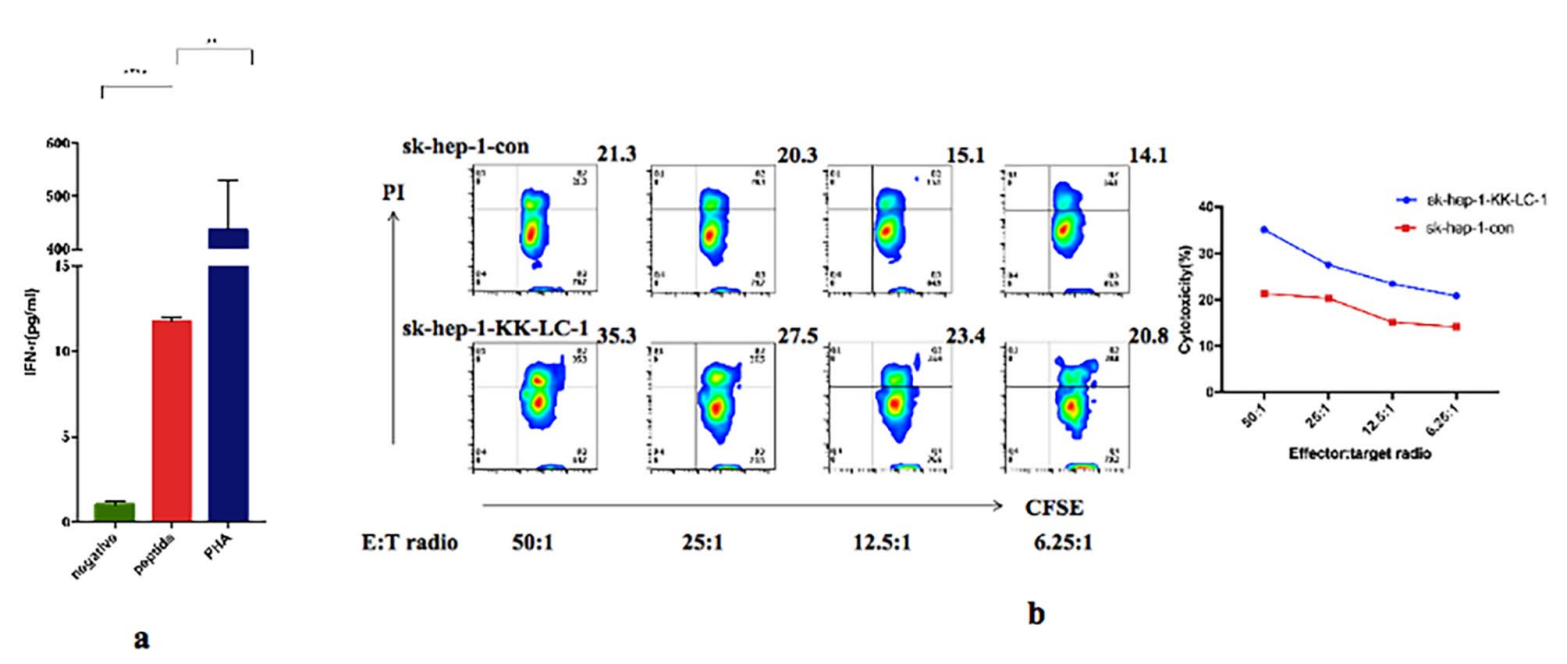
**a**: Antigenic peptides stimulate PBMCs. **b**: Validation of the cytotoxicity of reactive T cells against tumor cells with differential expression of KK-LC-1 in vitro

We stimulated and induced the secretion of γ-interferon (γ-IFN) of healthy human T cells with KK-LC-1 long peptide, and found that the γ-IFN secreted from T cells in serum was significantly increased after antigenic peptide stimulation (Fig. 4a).

We obtained reactive T cells by stimulating T cells with KK-LC-1 long peptide in vitro, and incubated them with different KK-LC-1 expressed tumor cells to observe the difference of killing effect. It was found that with the increase if potent target ratio, the killing efficiency of T cells from both types of cells gradually increased, and the killing ability of sk-hep-1-KK-LC-1 was significantly higher than that of sk-hep-1-con (Fig. 4b).

### Differential genes and related signaling pathways of HCC with different expression of KK-LC-1

**Fig. 5:**
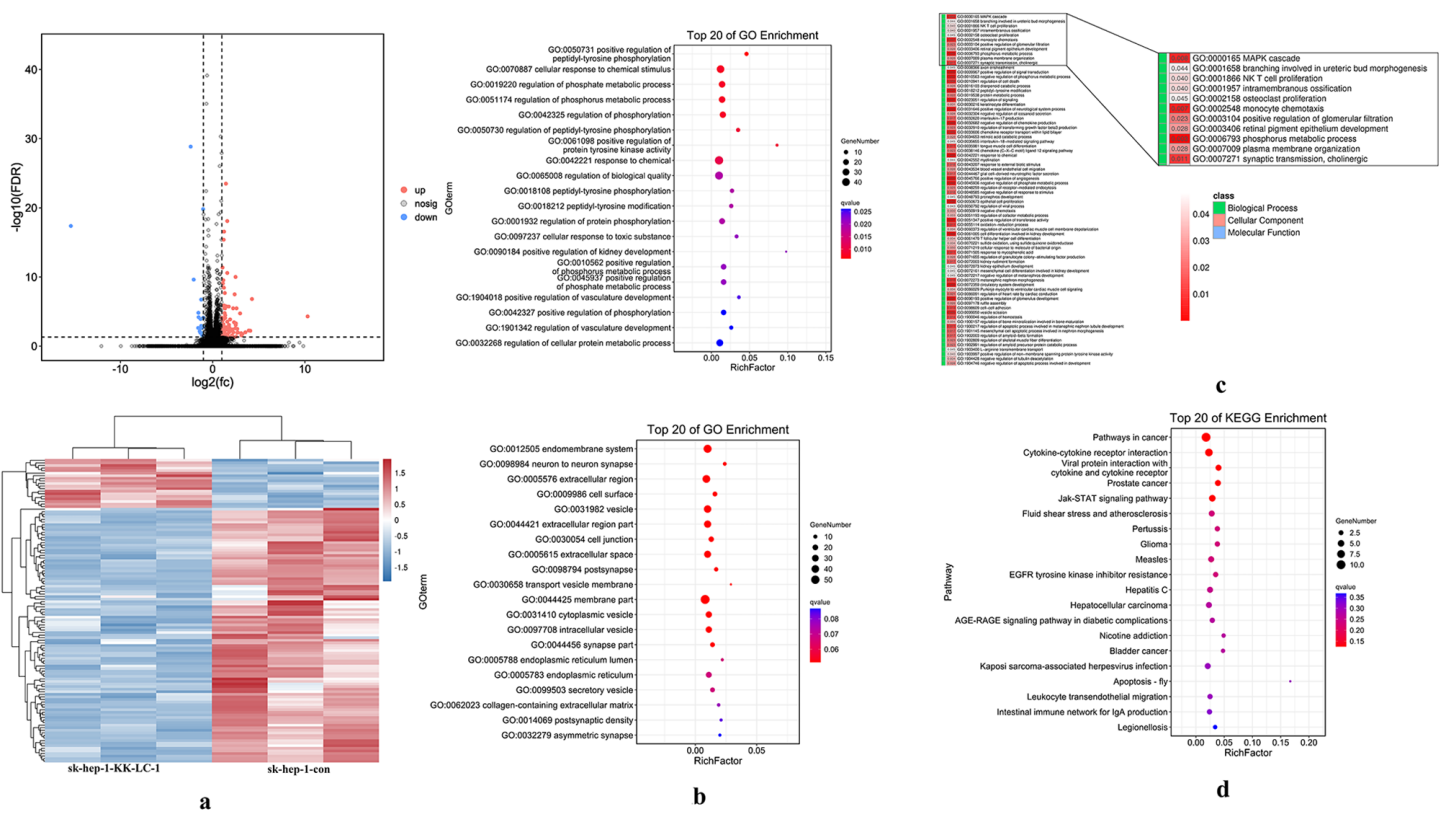
**a**: Differential genes of sh-hep-1-KK-LC-1 and sk-hep-1-con cells. **b**: GO pathway enriched by differential genes of sh-hep-1-KK-LC-1 and sk-hep-1-con cells. **c**: Differential genes enriched in MAPK signaling pathway. **d**: KEGG pathway enriched by differential genes of sh-hep-1-KK-LC-1 and sk-hep-1-con cells

By gene expression analysis, 118 genes with significantly differential expression between sk-hep-1-KK-LC-1 cells and sk-hep-1-con cells were screened. Compared with sk-hep-1-con HCC cells, 99 genes were dramatically decreased and 19 genes were increased in sk-hep-1-KK-LC-1 cells (Fig. 5a). Including *TGFA* and *PDGFB* genes, both of which were significantly reduced in cells with high expression of KK-LC-1.

Gene Ontology (GO) enrichment analysis was performed based on 118 genes associated with KK-LC-1 expression (Fig. 5b). The results showed that the differential genes mainly enriched in the biological processes such as positive regulation of peptide-tyrosine phosphorylation, cellular responses to chemical stimuli, and regulation of phosphate metabolic processes, and in molecular functions such as binding, regulation of cellular processes, and protein binding. Significant enrichment differences were also found in MARP-related GO pathways such as MAPK activation, MAPK cascade regulation, MAPK cascade positive regulation, MARK cascade innate immune negative regulation, and hepatic immune regulation (Fig. 5c).

The Kyoto Encyclopedia of Genes and Genomes (KEGG) enrichment analysis was also performed to identify the most significant signaling pathways involved in the differential genes. A total of 20 KEGG pathways with the largest differences were screened and the corresponding scatter plots were created (Fig. 5d). The results showed that the differences between the two groups were mainly in tumor pathway, EGFR tyrosine kinase inhibitor resistance and other related pathways.

## Discussion

In this study, we investigated expression rate of KK-LC-1 in liver cancer and determined its clinical application value in liver cancer tissues. Meanwhile, we also identified the correlation between the expression of KK-LC-1 and Foxp3. However, we did not detect the correlation between tissue KK-LC-1 expression and patient prognosis, which is inconsistent with previous findings [21]. The possible reasons may be the small number of cases and the inconsistent treatment of patients in this study.

Therefore, we individually analyzed 24 patients who received immunotherapy, and the results showed that the both expression of tissue KK-LC-1 and Foxp3 were correlated with OS. Patients with high KK-LC-1 expression were more likely to benefit from immunotherapy. In addition, we also found that the expression of KK-LC-1 in serum was also associated with the prognosis of patients received immunotherapy. These results suggest that KK-LC-1 could be one of the biomarkers for predicting the prognosis of immunotherapy in patients with liver cancer. By analyzing the expression levels of KK-LC-1 in patients’ tumors, we can more accurately assess the sensitivity to immunotherapy and prognosis. Therefore, KK-LC-1 as a biomarker holds the promise to serve as a critical basis for guiding the selection of immunotherapy strategies, thereby achieving the goal of personalized treatment.

In order to explore the mechanism of KK-LC-1 expression in immunotherapy of liver cancer, we analyzed the differential genes and differential signaling pathways enrichment between high-expressed and low-expressed cell lines. We found that the differentially expressed genes between the two cell lines include *TGFA* and *PDGFB*, which jointly involved in the MAPK signaling and EGFR tyrosine kinase inhibitor resistance pathway. Studies showed that MAPK signaling pathway is associated with immune escape of tumors [22]. Inhibition of MAPK pathway can enhance the specific recognition of cytotoxic T lymphocytes (CTL) [23], enhance the ability of immune T cells to attack tumors, induces apoptosis markers, upregulate HLA molecules, reduce tumor immunosuppressive factors, and then initiate immunotherapy [24]. Therefore, cells with high expression of KK-LC-1 may inhibit the MAPK signaling pathway by downregulating *TGFA* and *PDGFB*, thus showing enhanced anti-tumor immune response. The correlation between KK-LC-1 and PD-L1 is also worth noting. PD-L1 is an immune checkpoint molecule, whose overexpression inhibits immune responses, leading to immune evasion by tumors. The association between the expression of KK-LC-1 and PD-L1 may indicate the response of tumors to immune therapy. Understanding the relationship between KK-LC-1 and PD-L1 can better predict patients’ response to immune therapy and provide guidance for personalized treatment. EGFR is a receptor tyrosine kinase inhibitor, and its overactivation is associated with tumor progression and immune evasion. Currently, EGFR expression is significantly associated with PD-L1 and has now been demonstrated to be an independent factor in regulating PD-L1 protein in lung cancer [25]. Also, EGFR signaling pathway can be a mediator of immune escape and promote the progression of neoplasms, also an influencing factor regarding the prognosis of immunotherapy [26]. As a cancer-testis antigen, KK-LC-1 is not expressed in normal tissues other than the testes, so we also speculated that KK-LC-1 may be a tumor-associated antigen. After immunotherapy, tumor cells with high expression of KK-LC-1 would then be more easily recognized and killed by the human immune system. Subsequently, the number of tumor cells (especially the cells with high expression of KK-LC-1) decreased, and the corresponding antibody in the serum also decrease. With the extension of treatment time, the tumor regression rate will gradually decrease. KK-LC-1 is intricately linked to various immune-related signals in the development of tumors. In addition, previous studies have suggested that KK-LC-1 may interact with presenilin-1, thereby promoting the Notch1/Hes1 pathway in HCC, accelerating its progression [21]. Further exploration of the relationship between KK-LC-1 and these signals can aid in understanding the tumor’s response to immune therapy, thereby providing a theoretical basis for personalized treatment.

Tumor patients exhibit significant individual differences in immune status, and the serum levels of tumor markers can be influenced by various factors. These factors may include the patient’s age, immune status, nutritional status, inflammation, or infection, among others, and may not entirely reflect the specific immune response to the tumor [27]. Therefore, solely relying on serum antibody levels may not comprehensively assess patients’ response to immunotherapy. In subsequent studies, we will conduct larger-scale and longer-term clinical research, collect more patient data for in-depth analysis, and combine other immune monitoring indicators to enhance the accurate evaluation of patients’ immune status. This will provide more precise guidance for personalized treatment. However, in vitro validation experiments also have certain limitations. For example, in vitro experiments cannot fully simulate the complexity of the in vivo tumor microenvironment, including factors such as cell interactions, extracellular matrix, and immune cell infiltration. Therefore, in vitro results may not completely reflect the in vivo situation. In vitro experiments are typically static, while tumor growth, immune infiltration, and treatment effects may change over time, making it difficult to simulate the dynamic changes in the in vivo tumor microenvironment. We will continue to construct animal tumor models in subsequent studies to further verify the relationship between KK-LC-1 and tumor occurrence, development, and the prognosis of immunotherapy through in vivo experiments.

Although this study includes many liver cancer patients, the number of patients receiving immunotherapy is small. In some cases, different combination therapies such as chemotherapy or targeted therapy were applied clinically, which may have an impact on the prognosis analysis. Subsequently, we will expand the sample size for further stratified analysis, and extend the follow-up period to confirm that the expression of KK-LC-1 can be used as an effective biomarker for predicting efficacy and prognosis of the liver cancer patients with immunotherapy. Through comprehensive evaluation of patients’ clinical characteristics, treatment responses, and prognosis, we further elucidate the mechanism of action of KK-LC-1 in immunotherapy for liver cancer [28]. Simultaneously, through in vitro and in vivo experiments, we will continue to investigate the role of KK-LC-1 in tumor immune evasion, T cell activation, and modulation of the tumor microenvironment, providing a theoretical basis for the development of new treatment strategies.

KK-LC-1 can not only be used as a biomarker for predicting tumor prognosis, but also as a cancer testis antigen. Due to the presence of a natural barrier, it can be an ideal target for anti-tumor vaccine therapy [29]. In addition to CAR-T [30]/TCR-T [31], vaccine-based immunotherapies for CTAs are currently being developed, including peptide vaccines [32] and mRNA vaccines [33]. Tumor vaccines work by stimulating the patient’s own immune system to generate an immune response, thereby enhancing immune reactions and suppressing tumor growth [34]. ICIs have also been demonstrated to have significant anti-tumor effects in multiple types of cancer [35]. Through in vitro experiments, we have found that T cells stimulated by KK-LC-1 long peptides exhibit greater cytotoxicity against tumor cells, confirming the immunogenicity of KK-LC-1 long peptides predicted and synthesized by the research team earlier. We will further investigate the in vivo anti-tumor effects of tumor vaccines targeting KK-LC-1, while also validating their anti-tumor efficacy and synergistic effects with PD-1 and other ICIs. This will further confirm that the tumor vaccine targeting KK-LC-1, when combined with immune checkpoint inhibitors, can more effectively activate the anti-tumor immune system, enhance T cell-mediated tumor killing effects, and improve treatment outcomes. This study confirmed the immunogenicity of KK-LC-1 long peptide predicted and synthesized by the team at an early stage. We will further validate its anti-tumor efficacy and its synergistic effects with ICIs such as PD-1. In addition, mRNA vaccine is currently a safer and more effective immunotherapy than traditional vaccines, and we will follow up with a more in-depth study on it against KK-LC-1.

## Conclusion

This study firstly reports the relationship between KK-LC-1 antigen and its corresponding antibodies in liver cancer and immunotherapy. We conclude that patients with high expressed KK-LC-1 can significantly benefit from immunotherapy. Furthermore, changes in serum KK-LC-1 antibody can dynamically respond to and predict the efficacy of patients receiving immunotherapy. This assay has the advantages of less damaging and high compliance of patients, and has a good prospect for clinical application. Moreover, KK-LC-1 can be a potential target for anti-tumor immunotherapy.

### Electronic supplementary material

Below is the link to the electronic supplementary material.

Supplementary Fig. 1. KK-LC-1 expression in sk-hep-1-KK-LC-1 and sh-hep-1-con cells. a: Left: sk-hep-1-KK-LC-1 cell IHC staining (×20). Right: sk-hep-1-con cell IHC staining (×20). b: Flow cytometric analysis of KK-LC-1 expression in untransfected sk-hep-1, sh-hep-1-con and sk-hep-1-KK-LC-1 cells.

Supplementary Fig. 2. Relationship between KK-LC-1 expression level and PFS in patients.

Supplementary Fig. 3. a: Relationship between Foxp3 expression levels and the PFS in patients. b: Relationship between KK-LC-1 expression levels and the PFS in patients. c: Relationship between KK-LC-1 expression level and the PFS in patients with HCC. d: Relationship between KK-LC-1 expression level and the OS in patients the PFS in patients with HCC. e: Relationship between Foxp3 expression level and the PFS in patients with HCC. f: Relationship between Foxp3 expression level and the OS in patients with HCC.

## Data Availability

The raw data supporting the conclusions of this article will be made available by the authors, without undue reservation.

## Abbreviations

KK-LC-1: Kita-Kyushu lung cancer antigen-1
SD: stable disease
ICIs: Immune checkpoint inhibitors
PD-1: programmed cell death receptor 1
PD-L1: programmed cell death-ligand 1
CTLA-4: Cytotoxic T lymphocyte associate protein-4
irAEs: immune-related adverse events
MSI-H: microsatellite instability-high
TMB: tumor mutation burden
CTA: Cancer-testis Antigen
TAA: tumor-associated antigen
FBS: fetal bovine serum
CBA: cytometric bead array
FDR: false discovery rate
GO: Gene ontology
KEGG: Kyoto Encyclopedia of Genes and Genomes
IHC: immunohistochemistry
HCC: hepatocellular carcinoma
OS: overall survival
PFS: progression free survival
PR: partial response
γ-IFN: γ-interferon
CTL: cytotoxic T lymphocytes
