# Supplementary figures and images for: KK-LC-1, a biomarker for prognosis of immunotherapy for primary liver cancer
Source: BMC Cancer. 2024 Jul 7;24:811. doi: 10.1186/s12885-024-12586-y (PMC11229184; doi:10.1186/s12885-024-12586-y)

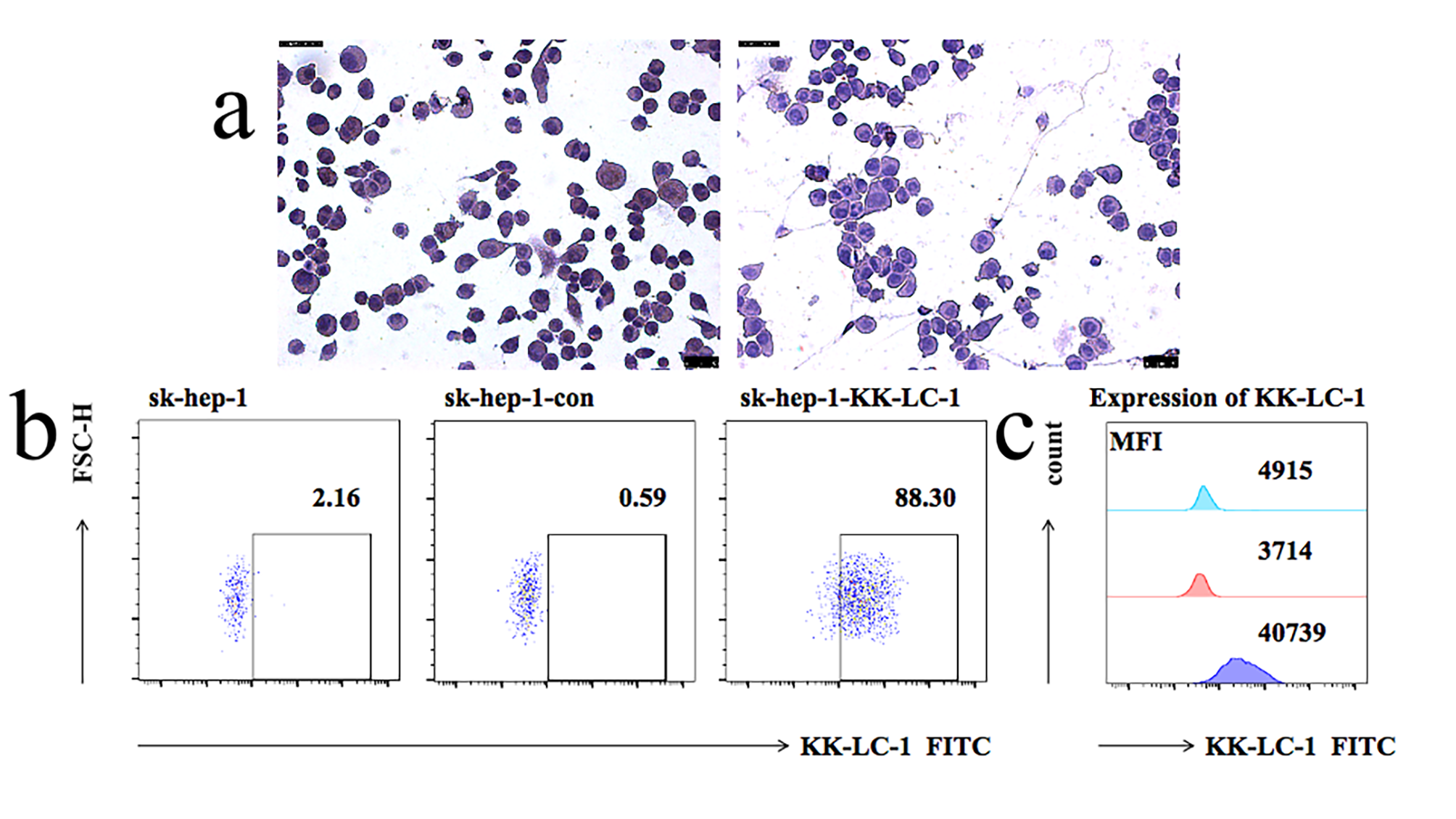

Supplement: Supplementary file 1 — Supplementary Fig. 1. KK-LC-1 expression in sk-hep-1-KK-LC-1 and sh-hep-1-con cells. a: Left: sk-hep-1-KK-LC-1 cell IHC staining (×20). Right: sk-hep-1-con cell IHC staining (×20). b: Flow cytometric analysis of KK-LC-1 expression in untransfected sk-hep-1, sh-hep-1-con and sk-hep-1-KK-LC-1 cells. [file 12885_2024_12586_MOESM1_ESM.png]

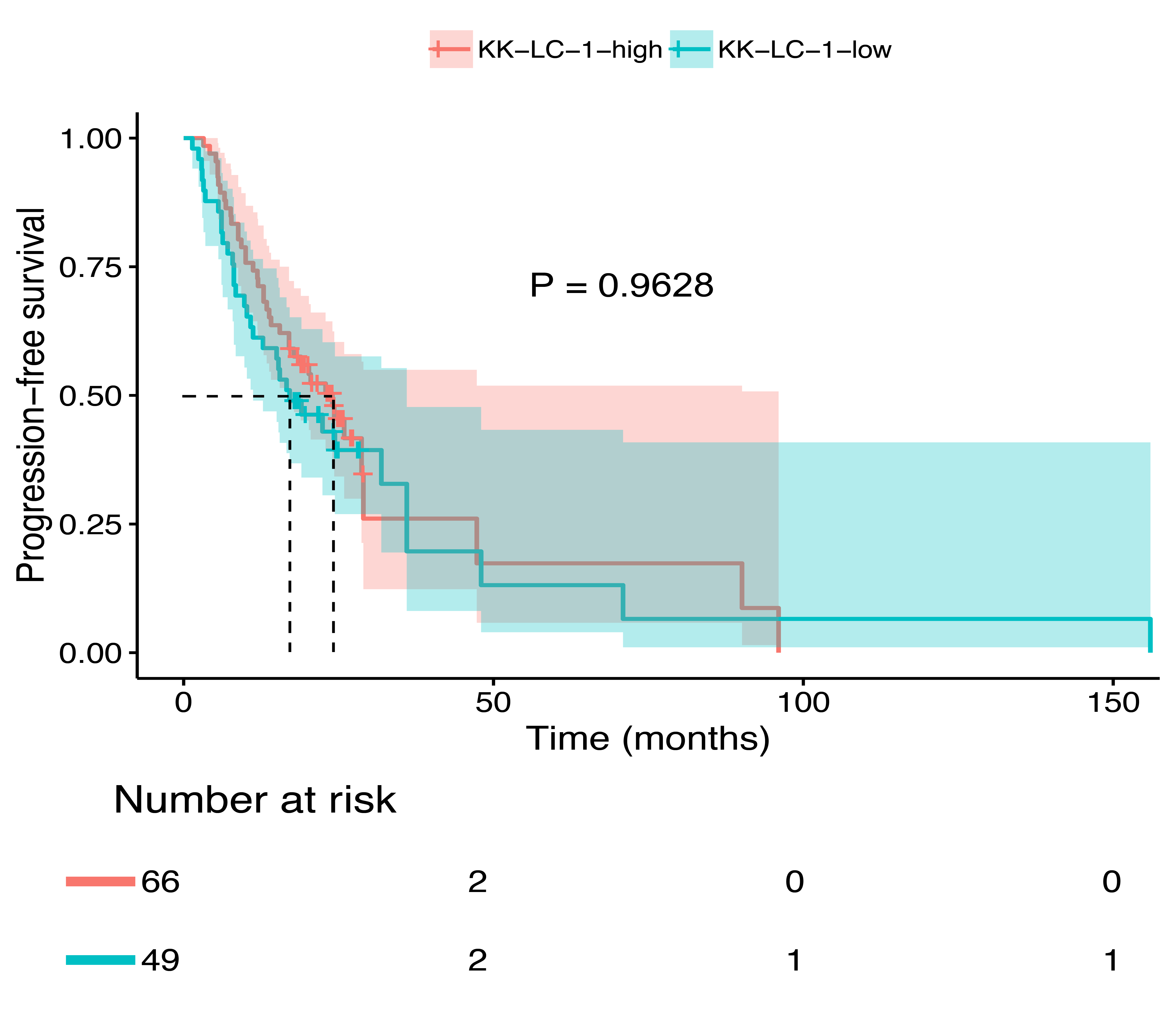

Supplement: Supplementary file 2 — Supplementary Fig. 2. Relationship between KK-LC-1 expression level and PFS in patients. [file 12885_2024_12586_MOESM2_ESM.png]

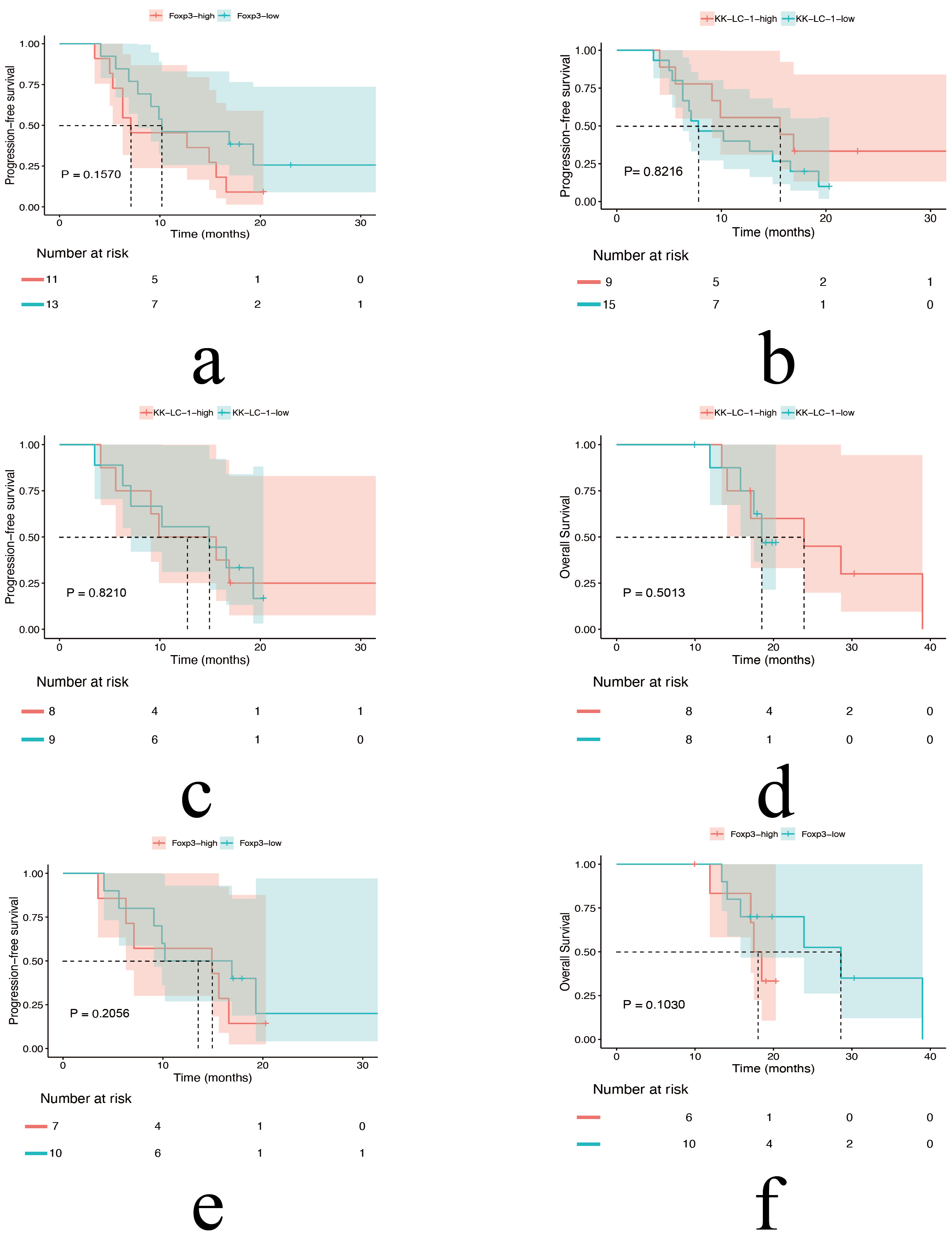

Supplement: Supplementary file 3 — Supplementary Fig. 3. a: Relationship between Foxp3 expression levels and the PFS in patients. b: Relationship between KK-LC-1 expression levels and the PFS in patients. c: Relationship between KK-LC-1 expression level and the PFS in patients with HCC. d: Relationship between KK-LC-1 expression level and the OS in patients the PFS in patients with HCC. e: Relationship between Foxp3 expression level and the PFS in patients with HCC. f: Relationship between Foxp3 expression level and the OS in patients with HCC. [file 12885_2024_12586_MOESM3_ESM.png]
